# The impact of *ABCB1*, *CYP3A4/5* and *ABCG2* gene polymorphisms on rivaroxaban trough concentrations and bleeding events in patients with non-valvular atrial fibrillation

**DOI:** 10.1186/s40246-023-00506-3

**Published:** 2023-07-07

**Authors:** Tingting Wu, Shuyi Wu, Li Li, Jing Xiang, Na Wang, Wenjun Chen, Jinhua Zhang

**Affiliations:** 1 Department of Pharmacy, Fujian Maternity and Child Health Hospital College of Clinical Medicine for Obstetrics & Gynecology and Pediatrics, Fujian Medical University, #18 Daoshan Road, Fuzhou, 350001 China; 2 Department of Pharmacy, Guizhou Provincial People’s Hospital, Guiyang, China; 3 Department of Pharmacy, The Second Affiliated Hospital of Chongqing Medical University, Chongqing, China

**Keywords:** Rivaroxaban, Gene polymorphisms, Pharmacokinetics, Bleeding, Non-valvular atrial fibrillation

## Abstract

**Background:**

The influence of genetic factors on the pharmacokinetics and clinical outcomes of rivaroxaban in patients with non-valvular atrial fibrillation (NVAF) is poorly understood. This study aimed to explore the effects of *CYP3A4/5*, *ABCB1*, and *ABCG2* gene polymorphisms on the trough concentrations and the bleeding risk of rivaroxaban in NVAF patients.

**Patients and methods:**

This study is a prospective multicenter study. The patient's blood samples were collected to detect the steady-state trough concentrations of rivaroxaban and gene polymorphisms. We visited the patients regularly at month 1, 3, 6, and 12 to record bleeding events and medications.

**Results:**

A total of 95 patients were enrolled in this study, and 9 gene loci were detected. For the dose-adjusted trough concentration ratio (C_trough_/D) of rivaroxaban, the homozygous mutant type was significantly lower than wild type at *ABCB1* rs4148738 locus (TT vs. CC, *P* = 0.033), and the mutant type was significantly lower than the wild type at *ABCB1* rs4728709 locus (AA + GA vs. GG, *P* = 0.008). *ABCB1* (rs1045642, rs1128503), *CYP3A4* (rs2242480, rs4646437), *CYP3A5* (rs776746), and *ABCG2* (rs2231137, rs2231142) gene polymorphisms had no significant effect on the C_trough_/D of rivaroxaban. For the bleeding events, we found that there were no significant differences among genotypes of all gene loci.

**Conclusion:**

This study found for the first time that *ABCB1* rs4148738 and rs4728709 gene polymorphisms had a significant impact on the C_trough_/D of rivaroxaban in NVAF patients. *CYP3A4/5*, *ABCB1*, and *ABCG2* gene polymorphisms were not associated with the bleeding risk of rivaroxaban.

## Introduction

Compared with warfarin, direct oral anticoagulants (DOACs) have many advantages, including rapid onset of action, no food interaction, less drug interaction, predictable pharmacokinetics and pharmacodynamics, etc [1] . DOACs have gradually been widely used in clinical practice, especially the use rate of rivaroxaban, which rose from 0.13% to 13.87% from 2011 to 2014 [2]. Non-valvular atrial fibrillation (NVAF) is one of the most common arrhythmias, which can increase the risk of ischemic stroke by about five times [3]. Oral anticoagulation therapy is an effective means to prevent thromboembolism in patients with NVAF. Studies have shown that the safety and effectiveness of rivaroxaban in patients with NVAF are as good as warfarin [4]. Although rivaroxaban does not require routine testing, it has been found that there are large inter-individual differences in patients with NVAF [5, 6].

Rivaroxaban is eliminated by dual hepatic and renal channels, and approximately two-thirds of rivaroxaban is metabolized by hepatic cytochrome P450 (CYP450) to inactive metabolites, mainly by CYP3A4/5 (approximately 18%) and CYP2J2 (approximately 14%) [7, 8]. Rivaroxaban is not only a substrate for CYP3A4/5 and CYP2J2, but also a substrate for P-glycoprotein (P-gp) and breast cancer resistance protein (BCRP) efflux transporters, which are abundant in epithelial cells of organs such as liver, intestine and kidney and may affect the PK process of the drug [9, 10]. CYP3A4/5, CYP2J2, P-gp and BCRP play important roles in the transport and metabolism of rivaroxaban. While the ATP-binding cassette subfamily B member 1(*ABCB1*) gene encodes P-gp, the ATP-binding cassette superfamily G member 2 (*ABCG2*) gene encodes BRCP [9, 11]. Therefore genetic polymorphisms in *CYP3A4/5*, *CYP2J2*, *ABCB1* and *ABCG2* may affect their functions and alter the pharmacokinetics of rivaroxaban, thus having an impact on the safety and efficacy of the drug.

Interindividual genetic variations in drug-metabolizing enzymes and transporters influence the pharmacokinetics and pharmacodynamics of various drugs [12]. Studies have shown that the inter-individual differences of rivaroxaban may be related to the gene polymorphisms of related metabolic enzymes and transporters [13]. At present, there are limited studies of gene polymorphisms about rivaroxaban, and the results are contradictory. Therefore, it is necessary to explore the influence of genetic factors on the pharmacokinetics of rivaroxaban further. Previous studies mostly analyzed the relationship between the plasma concentrations and gene polymorphisms [14–16] or investigated the impact of single gene polymorphism on trough concentration and bleeding events [17]. Therefore, the purpose of this study is to explore the effects of multiple gene polymorphisms (*CYP3A4/5*, *ABCB1*, and *ABCG2*) on the plasma concentrations of rivaroxaban and bleeding events, which may provide references for rational clinical use of rivaroxaban and further exploration of the influence of genes on the pharmacokinetics and clinical outcomes of rivaroxaban.

## Methods

### Study design and participants

This is a prospective multicenter study. It was carried out from September 2018 to September 2020. Inclusion criteria: more than 18 years old; diagnosed as NVAF; receiving rivaroxaban anticoagulant therapy. Exclusion criteria: combined with CYP3A4 or P-gp inducers or inhibitors (dronedarone, verapamil, quinidine, ticagrelor, clarithromycin, erythromycin, HIV protease inhibitors, azole antifungal drugs, rifampicin, carbamazepine, phenobarbital, valproic acid, and phenytoin) [5], severe liver impairment (Child–Pugh B/C and liver cirrhosis) or renal dysfunction (estimated glomerular filtration rate [eGFR] < 30 ml/(min*1.73 m^2^; comorbidities with significant risk for bleeding such as peptic ulcer and thrombocytopenia, or clinically active bleeding; unable to fulfill the follow-up visits. Informed consent was obtained from all individual participants included in the study. The studies involving human participants were reviewed and approved by Fujian Medical University Union Hospital Ethics Committee.

The patients took rivaroxaban (10/15/20 mg once daily) for at least one week. And then, peripheral venous blood was collected within 30 min before administration in the morning to detect the steady-state trough plasma concentrations and gene polymorphisms. We visited the patients at months 1, 3, 6, and 12 and recorded the medications, bleeding events, etc.

### Determination of plasma concentrations

We used the validated high-performance liquid chromatography/tandem mass (HPLC–MS/MS) method to detect the plasma concentrations of rivaroxaban [18]. This method used rivaroxaban-d4 as the internal standard, and the blood samples were processed by the protein precipitation method. The running time was 6 min. The linear range of the calibration curve was 2–500 ng/mL, and the calibration curve showed good linearity with R = 0.9998. The method was fast, accurate, and sensitive.

### Determination of gene polymorphisms

This study investigated the gene polymorphisms at the following gene loci: *ABCB1* (rs1045642, rs1128503, rs4148738, and rs4728709), *CYP3A4* (rs2242480 and rs4646437), CYP3A5 (rs776746), and *ABCG2* (rs2231137 and rs2231142), a total of 9 gene loci. Gene polymorphisms were detected by Massarray SNP typing technology, completed by BGI Tech Solutions (Beijing Liuhe) co., Limited.

### Statistical analysis

The chi-square test evaluated whether the allele frequencies distribution of genes conformed to the Hardy–Weinberg equilibrium. Kruskal–Wallis test was used to compare continuous variables. Categorical variables were compared using the Chi-square or Fisher's Exact tests. Results with *P* < 0.05 were considered statistically significant. Statistical analysis was performed using SPSS 26.0.

One study reported that rivaroxaban clearance decreased with increasing renal impairment, leading to increased plasma exposure and increased pharmacodynamic effects [19]. Meanwhile, in the PPK study of rivaroxaban in NVAF patients, creatinine clearance, age and body weight were found to have a significant effect on the PK of rivaroxaban [6]. Amiodarone is a commonly used combination drug for the treatment of patients with atrial fibrillation and is a moderate CYP3A4 inhibitor and P-gp inhibitor [20]. Amiodarone has a minor effect on rivaroxaban blood levels [5]. However, A study also found an increased risk of bleeding in patients taking rivaroxaban in combination with amiodarone than without amiodarone [21]. The tools commonly used in clinical practice to assess the risk of bleeding and thrombosis in patients with non-valvular atrial fibrillation are the HAS-BLED and CHA2DS2-VASc score, respectively. To avoid the influence of patients' own bleeding and thrombotic risks on the results of this study, they were included as confounding factors. In summary, to exclude the influence of the above confounding factors on the plasma concentrations of rivaroxaban, we analyzed whether there were differences in the ratio of amiodarone, age, weight, creatinine clearance (CrCl), CHA2DS2-VASc scores, and HAS-BLED scores among genotypes.

## Results

We enrolled a total of 95 patients, including 56 males and 39 females, with an average age of 66 years. The patients mainly received rivaroxaban 15 mg once daily. The patient's baseline characteristics are shown in Table 1. The allele frequencies distribution of all genes conformed to the Hardy–Weinberg equilibrium (Table 2).

**Table 1:** Baseline characteristics of the patients

Variables	Characteristics of patients (95)
Age (years)	65.8 ± 12.5
Female, n (%)	39 (41.0)
Body weight (kg)	67.8 ± 12.2
CrCl (mL/min)	77.3 ± 23.4
Dose, n (%)	
10 mg qd	21 (22.1)
15 mg qd	71 (74.7)
20 mg qd	3 (3.2)

*CrCl* creatinine clearance, *qd* once daily

**Table 2:** Distribution of genotypes and allele frequencies

Gene	SNP	Genotype	n (%)	Allele	n (%)	Hardy–Weinberg equilibrium, *p* value
*ABCB1*	rs1045642	AA	15 (15.8)	A	80 (42.1)	0.438
		AG	50 (52.6)	G	110 (57.9)	
		GG	30 (31.6)			
	rs1128503	AA	35 (36.8)	A	120 (63.2)	0.202
		AG	50 (52.6)	G	70 (36.8)	
		GG	10 (10.5)			
	rs4148738	CC	15 (15.8)	C	81 (42.6)	0.342
		CT	51 (53.7)	T	109 (57.4)	
		TT	29 (30.5)			
	rs4728709	GG	66 (72.5)	G	155 (85.2)	0.998
		GA	23 (25.3)	A	27 (14.8)	
		AA	2 (2.2)			
*CYP3A4*	rs2242480	CC	39 (42.8)	C	123 (67.6)	0.220
		CT	45 (49.4)	T	59 (32.4)	
		TT	7 (7.7)			
	rs4646437	GG	60 (65.9)	G	148 (813)	0.903
		AG	28 (30.8)	A	34 (18.7)	
		AA	3 (3.3)			
*CYP3A5*	rs776746	TT	7 (8.0)	T	54 (30.7)	0.520
		TC	40 (45.4)	C	122 (69.3)	
		CC	41 (46.6)			
*ABCG2*	rs2231137	CC	42 (46.2)	C	125 (68.7)	0.652
		TC	41 (45.0)	T	57 (31.3)	
		TT	8 (8.8)			
	rs2231142	GG	45 (49.4)	G	127 (69.8)	0.732
		GT	37 (40.6)	T	55 (30.2)	
		TT	9 (9.9)			

### Impact of gene polymorphisms on plasma concentrations

A total of 9 single nucleotide polymorphisms (SNPs) were investigated in this study, including *ABCB1* (4 SNPs), *CYP3A4/5* (3 SNPs), and *ABCG2* (2 SNPs). The results showed that *ABCB1* SNP rs4148738 (*P* = 0.039) and SNP rs4728709 (*P* = 0.029) had a significant effect on the C_trough_/D of rivaroxaban **(**Table 3**)**. For *ABCB1* rs4148738, the results of multiple comparisons showed that the homozygous mutant type was associated with a significantly lower C_trough_/D than wild type (TT vs. CC, *P* = 0.033), while the C_trough_/D between the heterozygous mutant type and homozygous mutant type or wild type did not show a significant difference (CT vs. TT, *P* = 0.611; CT vs. CC, *P* = 0.242) (Table 4). For *ABCB1* rs4728709, the results of multiple comparisons showed that the homozygous mutant type had a significantly lower C_trough_/D than wild type (GA vs. GG, *P* = 0.032), while the C_trough_/D between the homozygous mutant type and wild type or heterozygous mutant type was similar (AA vs. GG, *P* = 1.00; AA vs. GA, *P* = 1.00) (Table 4). We tried to compare the mutant type (AA + GA) with wild type (GG), and the results showed that the mutant type had a significantly lower C_trough_/D of rivaroxaban than wild type (*P* = 0.008). The study did not find that *ABCB1* (rs1045642 and rs1128503), *CYP3A4* (rs2242480 and rs4646437), *CYP3A5* (rs776746), and *ABCG2* (rs2231137 and rs2231142) gene polymorphisms had a significant effect on the C_trough_/D of rivaroxaban.

**Table 3:** Comparison of the rivaroxaban C_trough_/D among genotypes

SNP	Genotype	C_trough_/D	*P*
*ABCB1* rs1045642 (A > G)	AA	1.34 (0.66,3.25)	0.072
	AG	0.89 (0.37,1.88)	
	GG	0.71 (0.34,1.11)	
*ABCB1* rs1128503 (A > G)	AA	0.92 (0.51,2.17)	0.082
	AG	0.97 (0.39,1.70)	
	GG	0.39 (0.21,1.00)	
*ABCB1* rs4148738 (C > T)	CC	1.22 (0.66,3.47)	0.039*
	CT	0.88 (0.38,1.84)	
	TT	0.66 (0.31,1.20)	
*ABCB1* rs4728709 (G > A)	GG	1.06 (0.50,1.81)	0.008*
	GA	0.40 (0.30,0.88)	
	AA		
*CYP3A4* rs2242480(C > T)	CC	0.96 (0.42,1.98)	0.505
	CT	0.71 (0.29,1.60)	
	TT	1.10 (0.63,2.19)	
*CYP3A4* rs4646437(G > A)	GG	0.81 (0.40,1.55)	0.591
	GA	0.81 (0.26,1.71)	
	AA	1.10 (1.03,-)	
*CYP3A5* rs776746(T > C)	TT	1.10 (0.63,2.19)	0.395
	TC	1.01 (0.50,1.73)	
	CC	0.66 (0.36,1.51)	
*ABCG2* rs2231137(C > T)	CC	0.78 (0.35,1.55)	0.419
	CT	1.00 (0.40,2.00)	
	TT	0.66 (0.21,1.33)	
*ABCG2* rs2231142(G > T)	GG	0.92 (0.36,1.55)	0.976
	GT	0.88 (0.43,1.78)	
	TT	0.83 (0.34,2.26)	

^*^The mark means that there is a statistical difference

**Table 4:** Pair comparison of Cthrough/D between ABCB1 rs4148738(C > T) and ABCB1 rs4728709 (G > A) genotypes

SNP	Genotype	Adjusted *P* value^a^
*ABCB1* rs4148738 (C > T)	CC-CT	0.242
	CC-TT	0.033*
	TT-CT	0.611
*ABCB1* rs4728709 (G > A)	AA-GG	1.000
	AA-GA	1.000
	GA-GG	0.032*

^a^Bonferroni correction has adjusted *P* values for multiple tests

*The mark means that there is a statistical difference

### Impact of gene polymorphisms on bleeding events

A total of 16 bleeding events were found in this study, including 9 gingival bleedings, 3 conjunctiva hemorrhages, 3 hematurias, and 1 epistaxis. The study found no significant differences in the incidences of bleeding events among genotypes of all SNPs (Table 5).

**Table 5:** Comparison of bleeding events among genotypes

SNP	Genotype	Bleeding	No-bleeding	*P*
*ABCB1* rs1045642 (A > G)	AA	1	14	0.609
	AG	10	40	
	GG	5	25	
*ABCB1* rs1128503 (A > G)	AA	4	31	0.484
	AG	11	39	
	GG	1	9	
*ABCB1* rs4148738 (C > T)^a^	CC	3	12	1.000
	CT	9	42	
	TT	4	25	
*ABCB1* rs4728709 (G > A)^a^	GG	13	53	0.581
	GA	3	20	
	AA	0	2	
*CYP3A4* rs2242480(C > T)	CC	6	33	0.822
	CT	9	36	
	TT	1	6	
*CYP3A4* rs4646437(G > A)^a^	GG	11	49	0.793
	GA	4	24	
	AA	1	2	
*CYP3A5* rs776746 (T > C)	TT	1	6	0.827
	TC	8	32	
	CC	6	35	
*ABCG2* rs2231137(C > T)	CC	6	36	0.244
	CT	7	34	
	TT	3	5	
*ABCG2* rs2231142(G > T)	GG	8	37	0.852
	GT	6	31	
	TT	2	7	

^a^Because it does not meet the requirements of the R*C chi-square test, the homozygous mutations and heterozygous mutations were combined for analysis

To exclude the influence of other confounding factors on the plasma concentrations of rivaroxaban, this study also analyzed and compared the ratio of amiodarone, age, weight, CrCl, CHA2DS2-VASc scores, and HAS-BLED scores among genotypes for all gene loci. The results showed that the above confounding factors were almost similar among genotypes, except for the significant difference in the ratio of amiodarone for *ABCG2* rs2231137 (Table 6).

**Table 6:** Comparison of confounding factors among genotypes

	Wild type	Heterozygotes mutant type	Homozygotes mutant type	*P*
*ABCB1* rs1045642 (A > G)	15	50	30	
Age	65.9 ± 13.2	66.8 ± 11.3	64.1 ± 14.2	0.641
Weight	65.4 ± 15.1	67.1 ± 10.9	70.4 ± 12.9	0.369
Amiodarone	3 (20%)	12 (24%)	6 (20%)	0.943
CrCl	75.5 ± 25.1	73.6 ± 23.6	85.4 ± 20.6	0.120
CHA2DS2-VASc	2.7 ± 2.0	2.6 ± 2.0	2.2 ± 1.8	0.555
HAS-BLED	1.6 ± 1.0	1.5 ± 1.1	1.2 ± 1.0	0.342
*ABCB1* rs1128503 (A > G)	35	50	10	
Age	66.6 ± 13.9	65.0 ± 11.4	67.3 ± 13.7	0.773
Weight	65.4 ± 12.8	70.4 ± 11.7	62.1 ± 10.4	0.063
Amiodarone	9 (25.7%)	9 (18.0%)	3 (30%)	0.515
CrCl	75.9 ± 24.4	78.0 ± 24.1	78.5 ± 16.4	0.914
CHA2DS2-VASc	2.8 ± 2.2	2.3 ± 1.7	2.5 ± 1.9	0.517
HAS-BLED	1.5 ± 1.1	1.4 ± 1.0	1.4 ± 1.1	0.983
*ABCB1* rs4148738 (C > T)	15	51	29	
Age	67.3 ± 13.5	66.7 ± 12.0	63.5 ± 12.9	0.479
Weight	65.1 ± 14.5	67.2 ± 11.8	70.3 ± 11.7	0.382
Amiodarone	1 (6.7%)	15 (29.4%)	5 (17.2%)	0.147
CrCl	71.3 ± 25.4	74.2 ± 23.2	86.7 ± 20.3	0.052
CHA2DS2-VASc	3.1 ± 2.0	2.5 ± 2.0	2.5 ± 1.9	0.349
HAS-BLED	1.7 ± 1.2	1.5 ± 1.0	1.1 ± 1.0	0.155
*ABCB1* rs4728709 (G > A)	66	23	2	
Age	65.6 ± 13.2	65.3 ± 11.5	66.0 ± 11.4	0.988
Weight	68.1 ± 12.5	70.3 ± 10.9	57.5 ± 3.5	0.344
Amiodarone	17 (25.8%)	3 (13.0%)	1 (50%)	0.324
CrCl	76.2 ± 23.5	84.8 ± 21.4	65.8 ± 10.5	0.253
CHA2DS2-VASc	2.7 ± 2.1	1.8 ± 1.6	2.5 ± 0.7	0.157
HAS-BLED	1.5 ± 1.1	1.0 ± 0.9	2 ± 0	0.096
*CYP3A4* rs2242480(C > T)	39	45	7	
Age	61.4 ± 11.8	64.5 ± 12.7	67.9 ± 12.5	0.301
Weight	65.4 ± 12.4	69.9 ± 13.5	67.2 ± 10.2	0.476
Amiodarone	9 (23.1%)	10 (22.2%)	2 (28.6%)	0.932
CrCl	80.0 ± 21.0	80.5 ± 21.0	75.2 ± 25.6	0.588
CHA2DS2-VASc	1.6 ± 1.0	2.2 ± 1.8	2.9 ± 2.1	0.133
HAS-BLED	1.1 ± 1.1	1.3 ± 1.1	1.5 ± 1.1	0.506
*CYP3A4* rs4646437(G > A)	60	28	3	
Age	66.0 ± 12.5	66.0 ± 13.0	56.0 ± 9.8	0.403
Weight	68.2 ± 11.1	69.0 ± 14.3	66.0 ± 12.3	0.911
Amiodarone	11 (18.3%)	9 (32.1%)	1(33.3%)	0.135
CrCl	77.8 ± 24.6	78.1 ± 19.8	85.3 ± 26.9	0.863
CHA2DS2-VASc	2.5 ± 2.1	2.4 ± 1.8	2.0 ± 1.0	0.908
HAS-BLED	1.4 ± 1.1	1.5 ± 1.1	1.4 ± 0	0.699
*CYP3A5* rs776746 (T > C)	7	40	41	
Age	67.1 ± 12.2	65.3 ± 13.9	66.8 ± 11.4	0.848
Weight	64.4 ± 11.3	70.8 ± 13.6	66.4 ± 10.3	0.182
Amiodarone	1 (14.3%)	7 (17.5%)	12 (29.3%)	0.502
CrCl	73.6 ± 14.8	77.8 ± 20.2	77.1 ± 25.7	0.908
CHA2DS2-VASc	2.0 ± 1.3	2.3 ± 1.8	2.8 ± 2.2	0.352
HAS-BLED	1.3 ± 1.1	1.4 ± 1.1	1.4 ± 1.0	0.932
*ABCG2* rs2231137(C > T)	42	41	8	
Age	65.9 ± 12.8	65.5 ± 12.5	65.5 ± 13.9	0.990
Weight	68.0 ± 11.4	68.9 ± 13.4	67.9 ± 8.6	0.940
Amiodarone	15 (35.7%)	4(9.8%)	2 (25.0%)	0.012*
CrCl	77.8 ± 21.0	76.7 ± 25.2	89.4 ± 22.0	0.459
CHA2DS2-VASc	2.0 ± 1.9	2.8 ± 2.0	2.8 ± 1.7	0.176
HAS-BLED	1.3 ± 1.2	1.5 ± 1.0	1.4 ± 0.7	0.750
*ABCG2* rs2231142(G > T)	45	37	9	
Age	66.5 ± 13.1	64.0 ± 11.6	68.4 ± 14.8	0.534
Weight	66.9 ± 11.5	70.2 ± 13.2	68.0 ± 9.5	0.486
Amiodarone	8 (17.8%)	10(27.0%)	3 (33.3%)	0.411
CrCl	79.2 ± 21.8	78.3 ± 25.6	72.6 ± 17.3	0.763
CHA2DS2-VASc	2.6 ± 2.0	2.3 ± 1.9	2.4 ± 1.9	0.726
HAS-BLED	1.4 ± 0.9	1.4 ± 1.2	1.6 ± 1.1	0.876

*The mark means that there is a statistical difference

## Discussion

This is the first study to comprehensively evaluate the effects of multiple gene polymorphisms on the steady-state trough concentration of rivaroxaban and bleeding events in patients with NVAF. This study found that *ABCB1* rs4148738 and rs4728709 gene polymorphisms were associated with the C_trough_/D of rivaroxaban for the first time, which may provide a reference for the exploration of the influence of genetic factors on the pharmacokinetics of rivaroxaban.

At present, there are limited studies on the relationship between CYP450 gene polymorphisms and rivaroxaban. Sychev et al. [22] reported a significant correlation between *CYP3A* activity and rivaroxaban peak and trough levels in patients with deep vein thrombosis. In contrast, no significant correlation between *CYP3A* activity and treatment parameters was found. Nakagawa et al. [14] found the C_trough_/D of rivaroxaban did not differ significantly among CYP3A5*3 rs776746 and CYP2J2*7 rs890293 genotypes. Another study reported the peak steady-state rivaroxaban concentration between the heterozygous mutant type and wild-type for *CYP3A4* rs35599367 and *CYP3A5* rs776746 did not show a statistically significant difference in patients undergoing total hip and knee replacement surgery [8]. Our study also did not find that *CYP3A4* (rs2242480, rs4646437) and *CYP3A5* (rs776746) gene polymorphisms had a significant effect on the C_trough_/D of rivaroxaban. As far as we know, this is the first study to report the correlation between CYP450 gene polymorphisms and bleeding events of rivaroxaban, but our study found no significant correlation. Although no significant *CYP450* gene locus for rivaroxaban has been found so far, CYP450 is an important metabolic enzyme of rivaroxaban, and the related studies are limited at present. Its gene polymorphism needs to be further studied.

The results of previously published studies on the relationship between gene polymorphisms and rivaroxaban are inconsistent. A case report showed that the *ABCB1* gene mutation (rs1045642 and rs2032582) might lead to the prolonged half-life of rivaroxaban, which was related to the bleeding in an elderly patient with atrial fibrillation [23]. But some vivo and vitro studies (cell experiments, healthy Caucasians, patients undergoing total hip and knee replacement surgery, and NVAF patients) reported no significant correlation between the *ABCB1* (rs1128503, rs1045642, rs2032582, and rs4148738) gene polymorphisms and inter-individual pharmacokinetic variability of rivaroxaban was found [8, 15, 16, 24, 25]. Our study investigated four *ABCB1* SNPs, and the results showed that *ABCB1* SNP rs4148738 and SNP rs4728709 had a significant effect on the C_trough_/D of rivaroxaban. However, *ABCB1* SNP rs1045642 and SNP rs1128503 had no significant effect on the C_trough_/D of rivaroxaban. As far as we know, only two studies have explored the relationship between gene polymorphisms and the bleeding risk of rivaroxaban. Wang et al. reported no significant correlation between *ABCB1* gene variation loci rs1045642, rs1128503, rs4148738 and bleeding events in patients with atrial fibrillation [17]. In a real-world study in 2021, *ABCB1* gene polymorphisms (rs1045642, rs1128503, rs2032582) were not associated with the bleeding risk rivaroxaban [26]. Our analysis also did not find that the *ABCB1* gene polymorphisms significantly impacted the bleeding events of rivaroxaban.

This study showed that the *ABCB1* rs4148738 homozygous mutant type (TT) was associated with a significantly lower rivaroxaban C_trough_/D than wild type (CC), which means that patients with the TT genotype may have a lower bleeding risk than the CC genotype. Dimatteo et al. found that the *ABCB1* rs4148738 mutant type showed 5% lower trough concentrations of dabigatran compared with the wild type [24]. Lähteenmäki et al. also reported that compared with rs4148738 CC genotype, CT + TT genotype was associated with a reduced bleeding risk of apixaban [26]. The *ABCB1* rs4148738 homozygous mutant type reduced the C_trough_/D of rivaroxaban, suggesting that thrombotic events may occur when other factors can reduce the plasma concentration rivaroxaban are combined, such as the combination with CYP450/P-gp inducers (rifampicin, phenytoin sodium, doxorubicin, etc.) [5].

Our study showed that the *ABCB1* rs4728709 heterozygous mutant type (GA) had a significantly lower C_trough_/D of rivaroxaban than wild type (GG). A study reported that the *ABCB1* rs4728709 mutation could significantly increase the clearance rate of dexamethasone [27]. Dexamethasone is also a substrate of P-gp. An increase in clearance rate means a decrease in plasma concentration. The result is consistent with ours. The above results suggest that it may be necessary to pay attention to whether patients with mutations at this gene locus will have insufficient anticoagulation, especially when combined with CYP3A4 or P-gp inducers.

Nakagawa et al. reported that the C_trough_/D of rivaroxaban in patients with AF did not differ significantly among *ABCG2* c.421C > A (rs2231142) genotypes [14]. But they did not examine the effect of *ABCG2* gene polymorphisms on the bleeding events of rivaroxaban. Tandia et al. reported that *ABCG2* rs2231137 gene polymorphism was associated with plasma concentrations of sorafenib [28]. Studies have also found that *ABCG2* rs2231142 genotypes GT + TT were associated with increased exposure to sunitinib in people with neoplasms and increased plasma concentrations of rosuvastatin compared to genotype GG [29, 30]. This is the first study to investigate the impact of *ABCG2* gene polymorphisms on rivaroxaban's pharmacokinetics and bleeding events simultaneously, and no significant effects were found. This result may provide references for future exploration of the influence of *ABCG2* gene polymorphisms on rivaroxaban's pharmacokinetics and clinical outcomes.

Overall, there are fewer studies on the relevance of gene polymorphisms on the pharmacokinetics of rivaroxaban to the clinical outcome of rivaroxaban. Most studies have focused on the *ABCB1* gene, and the findings are somewhat contradictory. A total of nine genetic loci related to rivaroxaban metabolizing enzymes and transporters were examined in this study. We hope that the results of this study will be useful for the subsequent exploration of the influence of genetic factors on the pharmacokinetics and clinical outcomes of rivaroxaban. On one hand, this study provides some reference for further exploring genetic factors in the construction of a model of bleeding with rivaroxaban in patients with NVAF, which should be applied to predict the risk of bleeding with rivaroxaban and reduce the occurrence of adverse effects in clinical practice. On the other hand, it provides a certain reference in constructing a PPK/PD model of rivaroxaban in patients with NVAF, and helps to individualize and rationalize the clinical use of drugs.

This study has several limitations. Firstly, when analyzing bleeding events, we found that three gene loci did not meet the requirements of the R*C chi-square test, including *ABCB1* rs4148738, rs4728709, and *CYP3A4* rs4646437. So we combined heterozygous mutant type with homozygous mutant type and compared them with wild type. Therefore, we could only compare whether there were differences in bleeding events between the mutant and wild types at these three gene loci, but not multiple comparisons among three genotypes. Secondly, to eliminate the influence of confounding factors, this study further compared the ratio of amiodarone, age, weight, CrCl, CHA2DS2-VASc scores, and HAS-BLED scores among genotypes. However, it was found that there were significant differences in the ratio of amiodarone among genotypes at *ABCG2* rs2231137. Amiodarone is an inhibitor of CYP3A4 and P-gp. Studies have reported that amiodarone has a minor effect on the plasma concentrations of rivaroxaban [5]. So it may affect the final interpretation of the results of *ABCG2* rs2231137 in this study. Finally, due to the small sample size of this study, a later study with a larger clinical sample size is needed to further validate the results.

In general, there are limited studies on the influence of gene polymorphisms on the pharmacokinetics of rivaroxaban, and there are fewer studies on the correlation between gene polymorphisms and the clinical outcomes of rivaroxaban. Most studies focus on the *ABCB1* gene, and the results are also contradictory. This study investigated 9 gene loci related to rivaroxaban metabolic enzymes and transporters. It is hoped that the results will be helpful to the subsequent exploration of the influence of genetic factors on the pharmacokinetics and clinical outcomes of rivaroxaban.

## Conclusions

This study suggests that the *ABCB1* rs4148738 and rs4728709 gene polymorphisms significantly impacted the trough concentration of rivaroxaban. *CYP3A4/5*, *ABCB1*, and *ABCG2* gene polymorphisms were not associated with rivaroxaban bleeding events, and the relationship between genetic polymorphisms and bleeding events needs to be further investigated verified by the large sample size.

## Data Availability

The datasets generated during and/or analysed during the current study are available from the corresponding author on reasonable request.
